# Species–area relationships and additive partitioning of diversity of native and nonnative herpetofauna of the West Indies

**DOI:** 10.1002/ece3.2511

**Published:** 2016-10-05

**Authors:** De Gao, Gad Perry

**Affiliations:** ^1^ Department of Natural Resources Management Texas Tech University Lubbock TX USA

**Keywords:** additive diversity partitioning, herpetofauna, species–area relationship, West Indies, *Z*‐values

## Abstract

To evaluate the regional biogeographical patterns of West Indian native and nonnative herpetofauna, we derived and updated data on the presence/absence of all herpetofauna in this region from the recently published reviews. We divided the records into 24 taxonomic groups and classified each species as native or nonnative at each locality. For each taxonomic group and in aggregate, we then assessed the following: (1) multiple species–area relationship (SAR) models; (2) *C*‐ and *Z*‐values, typically interpreted to represent insularity or dispersal ability; and (3) the average diversity of islands, among‐island heterogeneity, γ‐diversity, and the contribution of area effect toward explaining among‐island heterogeneity using additive diversity partitioning approach. We found the following: (1) SARs were best modeled using the Cumulative Weibull and Lomolino relationships; (2) the Cumulative Weibull and Lomolino regressions displayed both convex and sigmoid curves; and (3) the Cumulative Weibull regressions were more conservative than Lomolino at displaying sigmoid curves within the range of island size studied. The *Z*‐value of all herpetofauna was overestimated by Darlington (*Zoogeography: The geographic distribution of animals*, John Wiley, New York, 1957), and *Z*‐values were ranked: (1) native > nonnative; (2) reptiles > amphibians; (3) snake > lizard > frog > turtle > crocodilian; and (4) increased from lower‐ to higher‐level taxonomic groups. Additive diversity partitioning showed that area had a weaker effect on explaining the among‐island heterogeneity for nonnative species than for native species. Our findings imply that the flexibility of Cumulative Weibull and Lomolino has been underappreciated in the literature. *Z*‐value is an average of different slopes from different scales and could be artificially overestimated due to oversampling islands of intermediate to large size. Lower extinction rate, higher colonization, and more in situ speciation could contribute to high richness of native species on large islands, enlarging area effect on explaining the between‐island heterogeneity for native species, whereas economic isolation on large islands could decrease the predicted richness, lowering the area effect for nonnative species. For most of the small islands less affected by human activities, extinction and dispersal limitation are the primary processes producing low species richness pattern, which decreases the overall average diversity with a large among‐island heterogeneity corresponding to the high value of this region as a biodiversity hotspot.

## 1 Introduction

The species–area relationship (SAR; MacArthur & Wilson, 1967), one of the closest things to a rule in ecology, has great utility for assessing species diversity and conservation needs. The shape of SARs provides information about underlying ecological processes and has been used in theoretical explorations of island biogeography, macroecology, species abundance distribution, and neutral models (Gavin & Sibanda, 2012; Lomolino, 2000; Pueyo, 2006; Rybicki & Hanski, 2013). SARs have been instrumental in studying scale‐dependent and other effects on biodiversity (Gerstner, Dormann, Václavík, Kreft, & Seppelt, 2014; Turner & Tjørve, 2005). Comparing SARs among taxa and regions helps explore dispersal and origination histories (Knapp, 2005; Stark, Bunker, & Carson, 2006; Wilkinson, 2003). Finally, insights from SARs have been applied in optimal reserve design (Neigel, 2003), identifying biodiversity hotspots (Fattorini, 2007) and assessing the impact that habitat fragmentation exerts on diversity (Harcourt & Doherty, 2005). Due to its generality, ecologists and conservationists are interested in mathematical modeling of SARs to explore the scale‐dependent relationship based on the shape of the selected model (Guilhaumon, Mouillot, & Gimenez, 2010; Lomolino & Weiser, 2001; Simaiakis, Tjørve, Gentile, Minelli, & Mylonas, 2012). Although Power model (Arrhenius, 1921) and Exponential model (Gleason, 1922) are most commonly invoked, sigmoid models, which have received more and more attention recently (Lomolino, 2000; Lomolino & Weiser, 2001; Triantis, Guilhaumon, & Whittaker, 2012), are found to be applicable when the spatial range exceeds three orders of magnitude (Triantis et al., 2012).

Despite its broad applicability, there are several limitations of the SAR. First, for some organisms, samples from fixed areas are difficult to obtain, and species richness can only be expressed in units of sampling effort (Crist & Veech, 2006). Second, differences in the relative abundances of species are often ignored (Gotelli & Colwell, 2001). Third, as we demonstrate in this study, species are generally not homogeneously distributed among islands, and the heterogeneity in biodiversity within (α) and among (β) islands cannot be revealed by the SAR. What's more, β‐diversity among islands is only partly explained by island area; thereby, SAR alone cannot quantify how much of the total β‐diversity is due to area (β_area_) and how much is due to other factors (β_replace_; Golodets, Kige, & Sternberg, 2011; Zajac, Vozarik, & Gibbons, 2013). However, the comparison of the diversity within (α) and among (β) islands and the contributions made by area (β_area_) and other factors (β_replace_) are important for strategic conservation planning because a low α‐diversity with a high β‐diversity suggests that species assemblages are heterogeneous among islands and species are often endemic to individual islands, while a high α‐diversity with a low β‐diversity indicates that species assemblages are homogenous and species within each island are a subsample of the same species pool (Francisco‐Ramos & Arias‐González, 2013); a low β_area_ with a high β_replace_ suggests that factors such as in situ speciation and human introduction may have a greater role in influencing patterns of β‐diversity, while a high β_area_ with a low β_replace_ indicates that species richness varies in a more predictable manner determined by factors such as habitat diversity, carrying capacity, and extinction/immigration dynamics as envisioned by MacArthur and Wilson (1967), Lomolino & Weiser (2001), Triantis & Bhagwat (2011).

Area can only explain a portion of species richness. Other factors, such as competition, dispersal, colonization, and speciation, also play important roles in influencing species richness (Crist & Veech, 2006). The logarithmic transformation of the power function (log*S* = *C *+ *Z* log*A*) is thus often used to calculate the *C*‐ and *Z*‐values, which are generally thought to be indicators of isolation (Rosenzweig, 1995), dispersal ability (Simaiakis et al., 2012), anthropogenic influence (Ficetola & Padoa‐Schioppa, 2009), scale of sampling (Crawley & Harral, 2001), trophic rank (Holt, Lawton, Polis, & Martinez, 1999), energy or latitude (Storch, Evans, & Gaston, 2005), habitat diversity (Tjørve & Turner, 2009), species diversity (Nilsson, Bengtsson, & Ås, 1988), or abundance distribution (Tjørve, Kunin, Polce, & Tjørve, 2008). The *Z*‐value, which tends to be less variable, is more often used (Lomolino, 2000; Watling & Donnelly, 2006). MacArthur and Wilson (1967) stated that the range of insular *Z*‐values was 0.20–0.35. Rosenzweig (1995) later narrowed it to 0.25–0.33. These ranges have later become a standard reference for numerous studies, below which the low *Z*‐values are thought to be an indicator of recent island formation, small degree of island isolation, and strong dispersal ability; consequently, the reduction in area fails to lead to a significant loss of species (Drakare, Lennon, & Hillebrand, 2006; Simaiakis et al., 2012; Triantis, Sfenthourakis, & Mylonas, 2008).

But as the increase in study interests on small islands (Lomolino, 2000; Triantis et al., 2006) and the improvement of calculation methods were represented by the applications of piecewise regression and information theory (Dengler, 2010; Gentile & Argano, 2005; Matthews, Steinbauer, Tzirkalli, Triantis, & Whittaker, 2014), scholars have argued that the species–area pattern comprises of more than one SAR, whereby processes operating at different spatial scales lead to different *Z*‐values (Gao & Perry, 2016; Lomolino & Weiser, 2001; Losos & Schluter, 2000; Morrison, 2014; Rosenzweig, 2004). Lomolino and Weiser (2001) and Rosenzweig (2004) even proposed three biological scales of species–area curve with three corresponding dominant processes of species addition and *Z*‐values ranges: (1) Stochastic extinction forces structure insular communities on small islands with *Z*‐values ranging from 0.10 to 0.20; (2) extinction/immigration dynamics on islands of intermediate size with *Z*‐values ranging from 0.25 to 0.45; and (3) speciation on relatively large islands with *Z*‐values higher than 0.60. And this proposal has been proved by Gao and Perry (2016), who recently detected the small island effect (SIE) of herpetofauna of the West Indies using a three‐segment piecewise regression approach. Thereby, we hypothesize that the overall *Z*‐value is an average of the three *Z*‐values of different scales, and the *Z*‐value calculated from intermediate to large islands could lead to an overestimation of the overall *Z*‐value.

**Figure 1:**
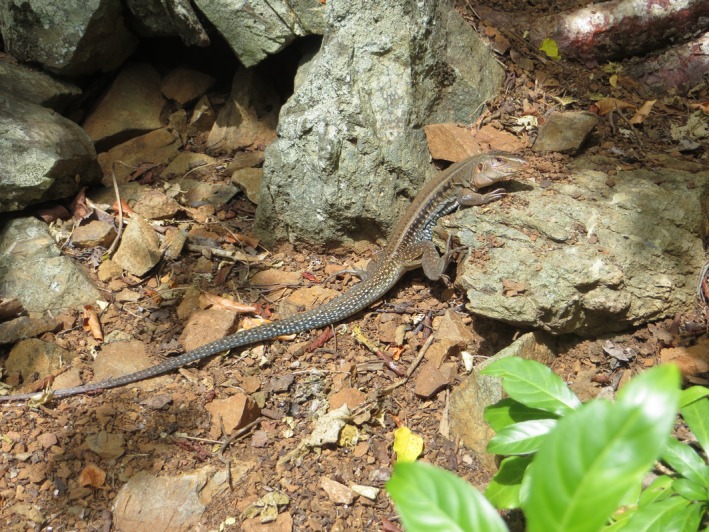
Puerto Rican ground lizard (*Ameiva exsul*) crawling on the ground, Guana Island of the British Virgin Islands. Photograph by De Gao, October 2013

The West Indies is a biodiversity hotspot (Myers, Mittermeier, Mittermeier, da Fonseca, & Kent, 2000), especially for amphibians and reptiles (Figure 1). Over 90% of the herpetofaunal species in the region are endemic, sometimes even to isolated areas within an island (Hedges, 2001). In order to reveal biogeographical patterns in this entire region, Darlington (1957) determined the SAR for the West Indian herpetofauna using only seven islands (Cuba, Hispaniola, Jamaica, Puerto Rico, Montserrat, Saba, and Redonda). Subsequently, various methods such as SAR (Losos, 1996; Ricklefs & Lovette, 1999; Wright, 1981), additive diversity partitioning (Francisco‐Ramos & Arias‐González, 2013), parsimony analysis of distributions (Trejo‐Torres & Ackerman, 2001), phylogenetic analysis (Rodríguez‐Robles, Jezkova, & García, 2007; Seal, Kellner, Trindl, & Heinze, 2011), chromosomal and genetic characterization (Giannoulis et al., 2014), ecomorphological evolution (Brandley & de Queiroz, 2004), and fossil evidence (Hall, Robbins, & Harvey, 2004) were used for many taxa.

However, there are several imperfections in previous studies concerning SAR. First, small islands are ignored, and most of these studies covered only a portion of the region, fewer than 200 islands in each case. Second, the Power model is used by default, without the evaluation of other possible candidate models. Third, the comparison of the diversity within and among islands and the contributions made by area and other factors remain blank. Fourth, although humans now become a major geological and environmental force (Corlett, 2015), studies for nonnative species are still insufficient. Here, we evaluate biogeographical patterns of native and nonnative herpetofauna in the West Indies, on a scale not previously attempted, comparing (1) SAR models, (2) *Z*‐values, and (3) the heterogeneity in biodiversity within and among islands. The aims of the study were to investigate the following: (1) Whether sigmoid instead of convex models provide a better performance; (2) whether the *Z*‐value is overestimated in previous studies and how *Z*‐values vary among groups; and (3) whether area plays the same role toward explaining the among‐island heterogeneity for native and nonnative species.

## 2 Methods

### 2.1 Study area and data

**Figure 2:**
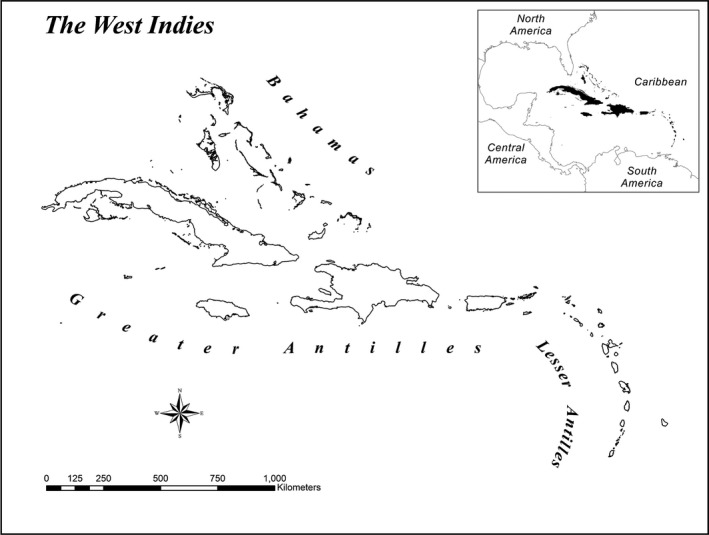
Map of the West Indies, showing the distribution of 1,668 studied islands

The West Indies comprises over 3,000 islands, cays, and emergent rocks belonging to three main island groups: Bahamas, Greater Antilles, and Lesser Antilles. We derived complete herpetological species lists for each island from Powell and Henderson (2012), who recorded more than 1,000 species on 749 islands. We digitized islands using base maps in ArcMap 10 and ArcGlobe 10 (ESRI, Redlands, CA, USA), including not only the 749 islands included in Powell and Henderson (2012) but also hundreds of small explored islands that have no herpetofaunal species, for a total of 1,668 islands varying in area by over 10 orders of magnitude, from 3.9 × 10^−5^ km^2^ to 1.1 × 10^5^ km^2^ (Figure 2). The resulting map was projected by a UTM_18N coordinate system with WGS_1984 datum.

### 2.2 Species–area relationship

The species records were classified into 24 taxonomic groups: all species, all native species, all nonnative species, all amphibian, amphibian native, amphibian nonnative, all reptile, reptile native, reptile nonnative, frog, frog native, frog nonnative, turtle, turtle native, turtle nonnative, lizard, lizard native, lizard nonnative, snake, snake native, snake nonnative, crocodilian, crocodilian native, and crocodilian nonnative. Power and Exponential are by far the best known models, whereof Power model is the most frequently applied to species–area data in the literature. Recent work indicates that the shape of SAR curves in arithmetic space is often sigmoid rather than convex and has an upper asymptote, and thus attempts to model the SARs using various functions have emerged. Dengler (2009) listed as many as 23 possible models, among which eight are commonly used and summarized by Guilhaumon et al. (2010). We investigated eight candidate models (Power, Exponential, Negative exponential, Monod, Rational, Logistic, Lomolino, and Cumulative Weibull), including both convex and sigmoid models, to analyze SARs in each group. The Akaike's information criterion (AIC) was applied as a criterion for model selection (Burnham & Anderson, 2002). We also calculated Akaike weights (ω) for all models to evaluate each model's probability of providing the best explanation of the data. Moreover, we calculated the first and second derivatives of the Lomolino and Cumulative Weibull to test the shape of the maximum‐likelihood curves fit for each taxonomic group, and we also applied a bootstrapping approach which resampled the data points 10,000 times and gave bootstrapped estimates of parameters and the probability that inflexion point occurs within the range of observed island size.

We also analyzed the linear function of the Power model (log*S*/log*A*) to compare *Z*‐values among different species groups and compare the *Z*‐value of all herpetofauna obtained here with that calculated by Darlington (1957). Because the logarithm of zero is undefined, and any data transformation such as log(*S *+* *1) biases *C*‐ and *Z*‐values when comparing among studies (Williamson, 1981). To avoid biasing the parameters, only islands with recorded species were analyzed. Although removing observations is not favored, we believe sacrificing some data in exchange for an unbiased result was worthwhile (Russell, Clout, & McArdle, 2004).

### 2.3 Additive diversity partitioning

Alpha diversity is the diversity of a single island or location; β‐diversity is the number of species in a region that are not observed on an island due to multiple localities, and γ‐diversity is the total diversity of a region. Because typically α‐diversity is measured with respect to some fixed spatial scales, rather than a scale that varies by several orders of magnitude, we replaced α‐diversity with the average diversity of islands in our dataset (hereafter referred to as “*a*” for brevity) and β‐diversity with among‐island heterogeneity (hereafter referred to as “*b*” for brevity). We used additive diversity partitioning to quantify the heterogeneity in biodiversity by comparing the diversity within (*a*) and among (*b*) islands and by comparing the contributions made by area (*b*_area_) and other factors (*b*_replace_). In the additive approach, diversity can be explored across spatial scales (Gering & Crist, 2002), and γ‐diversity (regional scale) is partitioned into the sum of the average diversity of islands (*a*) and among‐island heterogeneity (*b*). Because *a*,* b*, and γ‐diversity are measured using the same units, their relative importance can be quantified (Crist & Veech, 2006).

Although understanding the spatial scale at which diversity is generated is important, we also must know the reasons that cause one or more species to be missing from an island. When a species is missing from an island, one reason might be that the island is not large enough. So, we used additive diversity partitioning combined with SAR to partition *b* into *b*_area_, which represents the average difference between *a* and the diversity predicted for the largest sample (*S*_max_) and *b*_replace_, the average number of missing species that are not explained by area.

In order to unify the calculation method, we estimated *S*_max_ using the Lomolino model (we use Lomolino because Cumulative Weibull did not provide parameter estimation for nonnative crocodilians) for each group.

We performed all analyses using R 2.15.2 (R Development Core Team 2012) and used the mmSAR package (Guilhaumon et al., 2010) for modeling the SARs.

## 3 Results

**Figure 3:**
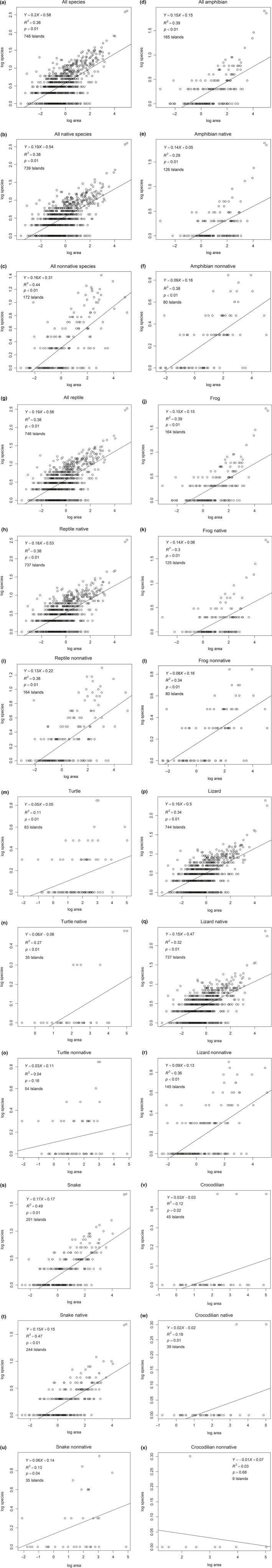
Base‐10 log‐transformed linear regression, showing the relationship between herpetofaunal species richness and island area (km^2^) for each taxonomic group. (a) All species. (b) All native species. (c) All nonnative species. (d) Amphibian. (e) Amphibian native. (f) Amphibian nonnative. (g) Reptile. (h) Reptile native. (i) Reptile nonnative. (j) Frog. (k) Frog native. (l) Frog nonnative. (m) Turtle. (n) Turtle native. (o) Turtle nonnative. (p) Lizard. (q) Lizard native. (r) Lizard nonnative. (s) Snake. (t) Snake native. (u) Snake nonnative. (v) Crocodilian. (w) Crocodilian native. (x) Crocodilian nonnative

Species richness increased as island area increased (Figure 3), and the positive SARs for the nonnative are consistent with Helmus, Mahler, and Losos (2014). A significant positive correlation (*p *<* *.05) existed between island area and the number of herpetofaunal species, except for the turtle nonnative (*p *=* *.16) and crocodilian nonnative (*p *=* *.68) groups (Figure 3). In the nonnative groups, extremely large values of species richness sometimes existed in small‐area islands and extremely small values of species richness existed in large‐area islands (Figure 3). Further, species–area plots of the native groups were more convergent to the regression line than those of the nonnative groups.

Species richness showed a phase of rapid rise across small‐island areas followed by a subsequent flattening toward an asymptote. The model with lower AIC value indicates a stronger evidence for being better over the others. However, according to Burnham and Anderson (2002), models with AIC differences (∆AIC) between zero and two have equal support, whereas models that differ from the best model by between four and seven have limited support and those that differ from the best model by 10 or more have no support. So, in our study, the model with the lowest AIC and models with ∆AIC no higher than two were all considered equally the best; for example, for amphibian species group (Table 1), the second best‐fit model had a ∆AIC of 30, which was >2, so only one relationship (Cumulative Weibull) was selected for this group. However, for all species and all nonnative species groups, the second best‐fit model had a ∆AIC ≤ 2, so two relationships were considered equally for these two groups. Exponential and Monod were never selected as best models; Negative exponential was selected only once; Rational and Logistic were selected three times; and Power was selected five times. The two most popular models were Cumulative Weibull (12 times) and Lomolino (eight times; Table 1).

**Table 1:** Evaluation of all candidate models based on the Akaike's information criterion values (AIC), the difference between the AIC values (∆AIC), the Akaike weights (ω), and the rank of functions (*r*) for each taxonomic group

Name		Code		Formula		Number of parameters		Shape		Asymptote		All species			
												AIC	∆AIC	*ω*	*r*
Power		Power		*S = cA* ^*z*^		2		Convex		No		4752.0	0.0	0.63	1
Exponential		Expo		*S = c + zlog(A)*		2		Convex		No		8746.7	3994.7	0.00	8
Negative exponential		Negexpo		*S = c(1−exp(−zA))*		2		Convex		Yes		5374.0	622.0	0.00	5
Monod		Monod		*S = c/(1 + zA* ^*−1*^ *)*		2		Convex		Yes		5448.0	696.0	0.00	7
Rational function		Ratio		*S = (c + zA)/(1 + fA)*		3		Convex		Yes		5332.8	580.8	0.00	4
Logistic		Logist		*S = c/(1 + exp(−zA + f))*		3		Sigmoid		Yes		5408.8	656.8	0.00	6
Lomolino		Lomolino		*S = c/(1 + z* ^*log(f/A)*^ *)*		3		Sigmoid/Convex		Yes		4755.0	3.0	0.14	3
Cumulative Weibull		Weibull		*S = c(1−exp(−zA* ^*f*^ *))*		3		Sigmoid/Convex		Yes		4754.0	2.0	0.23	2
**All native**				**All nonnative**				**All amphibian**				**Amphibian native**			
**AIC**	**∆AIC**	***ω***	***r***	**AIC**	**∆AIC**	***ω***	***r***	**AIC**	**∆AIC**	***ω***	***r***	**AIC**	**∆AIC**	***ω***	***r***
4415.3	0.0	0.61	1	1343.8	220.7	0.00	6	−601.4	94.4	0.00	3	−1162.5	416.7	0.00	6
8627.0	4211.7	0.00	8	1661.3	538.2	0.00	7	3422.1	4117.9	0.00	8	3220.4	4799.6	0.00	8
4741.0	325.7	0.00	6	1144.7	21.6	0.00	4	−574.4	121.4	0.00	5	−1560.9	18.3	0.00	2
4754.9	339.6	0.00	7	1976.6	853.5	0.00	8	696.7	1392.5	0.00	7	−223.2	1356.0	0.00	7
4701.3	286.0	0.00	4	1123.1	0.0	0.60	1	−591.2	104.6	0.00	4	−1480.3	98.9	0.00	3
4702.8	287.5	0.00	5	1204.7	81.6	0.00	5	−169.2	526.6	0.00	6	−1383.2	196.0	0.00	5
4417.9	2.6	0.17	3	1124.6	1.5	0.29	2	−665.8	30.0	0.00	2	−1409.3	169.9	0.00	4
4417.3	2.0	0.22	2	1126.5	3.4	0.11	3	−695.8	0.0	0.99	1	−1579.2	0.0	1.00	1
**Amphibian nonnative**				**All reptile**				**Reptile native**				**Reptile nonnative**			
**AIC**	**∆AIC**	***ω***	***r***	**AIC**	**∆AIC**	***ω***	***r***	**AIC**	**∆AIC**	***ω***	***r***	**AIC**	**∆AIC**	***ω***	***r***
−2510.0	216.3	0.00	6	4357.8	0.0	0.67	1	4075.4	75.5	0.00	2	343.6	193.3	0.00	6
−2198.6	527.7	0.00	7	8021.9	3664.1	0.00	8	7917.3	3917.4	0.00	8	620.8	470.5	0.00	7
−2693.1	33.2	0.00	4	4937.1	579.3	0.00	6	4419.3	419.4	0.00	6	167.7	17.4	0.00	4
−1979.9	746.4	0.00	8	4993.0	635.2	0.00	7	4449.0	449.1	0.00	7	918.1	767.8	0.00	8
−2718.4	7.9	0.01	3	4880.7	522.9	0.00	5	3999.9	0.0	1.00	1	150.3	0.0	0.77	1
−2621.0	105.3	0.00	5	4872.2	514.4	0.00	4	4311.7	311.8	0.00	5	213.6	63.3	0.00	5
−2726.3	0.0	0.63	1	4362.1	4.3	0.08	3	4077.7	77.8	0.00	4	155.1	4.8	0.07	3
−2725.2	1.1	0.36	2	4359.8	2.0	0.25	2	4077.4	77.5	0.00	3	153.5	3.2	0.16	2

Name		Code		Formula		Number of parameters		Shape		Asymptote		Frog			
												AIC	∆AIC	*ω*	*r*
Power		Power		*S = cA* ^*z*^		2		Convex		No		−615.8	97.0	0.00	3
Exponential		Expo		*S = c + zlog(A)*		2		Convex		No		3421.3	4134.1	0.00	8
Negative exponential		Negexpo		*S = c(1−exp(−zA))*		2		Convex		Yes		−591.2	121.6	0.00	5
Monod		Monod		*S = c/(1 + zA* ^*−1*^ *)*		2		Convex		Yes		689.2	1402.0	0.00	7
Rational function		Ratio		*S = (c + zA)/(1 + fA)*		3		Convex		Yes		−607.3	105.5	0.00	4
Logistic		Logist		*S = c/(1 + exp(−zA + f))*		3		Sigmoid		Yes		−181.4	531.4	0.00	6
Lomolino		Lomolino		*S = c/(1 + z* ^*log(f/A)*^ *)*		3		Sigmoid/Convex		Yes		−682.3	30.5	0.00	2
Cumulative Weibull		Weibull		*S = c(1−exp(−zA* ^*f*^ *))*		3		Sigmoid/Convex		Yes		−712.8	0.0	1.00	1
**Frog native**				**Frog nonnative**				**Turtle**				**Turtle native**			
**AIC**	**∆AIC**	***ω***	***r***	**AIC**	**∆AIC**	***ω***	***r***	**AIC**	**∆AIC**	***ω***	***r***	**AIC**	**∆AIC**	***ω***	***r***
−1143.0	412.1	0.00	6	−2639.3	213.4	0.00	6	−3507.6	259.9	0.00	6	−6540.2	8.0	0.01	3
3227.5	4782.6	0.00	8	−2329.3	523.4	0.00	7	−3084.0	683.5	0.00	7	−5680.4	867.8	0.00	8
−1542.0	13.1	0.00	2	−2822.8	29.9	0.00	4	−3759.6	7.9	0.02	2	−6299.6	248.6	0.00	5
−232.2	1322.9	0.00	7	−2111.4	741.3	0.00	8	−2906.4	861.1	0.00	8	−6073.8	474.4	0.00	7
−1462.1	93.0	0.00	3	−2846.8	5.9	0.04	3	−3757.1	10.4	0.01	3	−6371.8	176.4	0.00	4
−1362.1	193.0	0.00	5	−2762.1	90.6	0.00	5	−3648.6	118.9	0.00	5	−6134.6	413.6	0.00	6
−1439.0	116.1	0.00	4	−2852.7	0.0	0.85	1	−3756.9	10.6	0.00	4	−6548.2	0.0	0.58	1
−1555.1	0.0	1.00	1	−2848.6	4.1	0.11	2	−3767.5	0.0	0.97	1	−6547.5	0.7	0.41	2
**Turtle nonnative**				**Lizard**				**Lizard native**				**Lizard nonnative**			
**AIC**	**∆AIC**	***ω***	***r***	**AIC**	**∆AIC**	***ω***	***r***	**AIC**	**∆AIC**	***ω***	***r***	**AIC**	**∆AIC**	***ω***	***r***
−3524.4	205.8	0.00	6	3950.1	121.9	0.00	3	3819.9	328.4	0.00	3	−1999.0	75.4	0.00	6
−3441.7	288.5	0.00	7	6734.9	2906.7	0.00	8	6631.2	3139.7	0.00	8	−1633.2	441.2	0.00	7
−3730.2	0.0	0.66	1	4157.8	329.6	0.00	6	3887.2	395.7	0.00	6	−2020.0	54.4	0.00	4
−3275.3	454.9	0.00	8	4254.9	426.7	0.00	7	4234.8	743.3	0.00	7	−1304.9	769.5	0.00	8
−3707.9	22.3	0.00	3	3838.3	10.1	0.01	2	3586.1	94.6	0.00	2	−2057.1	17.3	0.00	3
−3702.1	28.1	0.00	5	3828.2	0.0	0.99	1	3491.5	0.0	1.00	1	−2002.1	72.3	0.00	5
−3706.6	23.6	0.00	4	3955.4	127.2	0.00	5	3823.2	331.7	0.00	5	−2074.4	0.0	0.97	1
−3728.9	1.3	0.34	2	3952.1	123.9	0.00	4	3822.5	331.0	0.00	4	−2067.1	7.3	0.03	2

Name		Code		Formula		Number of parameters		Shape		Asymptote		Snake			
												AIC	∆AIC	*ω*	*r*
Power		Power		*S = cA* ^*z*^		2		Convex		No		−546.8	0.0	0.60	1
Exponential		Expo		*S = c + zlog(A)*		2		Convex		No		2113.4	2660.2	0.00	8
Negative exponential		Negexpo		*S = c(1−exp(−zA))*		2		Convex		Yes		323.6	870.4	0.00	5
Monod		Monod		*S = c/(1 + zA* ^*−1*^ *)*		2		Convex		Yes		689.1	1235.9	0.00	7
Rational function		Ratio		*S = (c + zA)/(1 + fA)*		3		Convex		Yes		155.5	702.3	0.00	4
Logistic		Logist		*S = c/(1 + exp(−zA + f))*		3		Sigmoid		Yes		490.7	1037.5	0.00	6
Lomolino		Lomolino		*S = c/(1 + z* ^*log(f/A)*^ *)*		3		Sigmoid/Convex		Yes		−544.5	2.3	0.19	3
Cumulative Weibull		Weibull		*S = c(1−exp(−zA* ^*f*^ *))*		3		Sigmoid/Convex		Yes		−544.7	2.1	0.21	2
**Snake native**				**Snake nonnative**				**Crocodilian**				**Crocodilian native**			
**AIC**	**∆AIC**	***ω***	***r***	**AIC**	**∆AIC**	***ω***	***r***	**AIC**	**∆AIC**	***ω***	***r***	**AIC**	**∆AIC**	***ω***	***r***
−874.5	0.0	0.59	1	−3029.9	115.0	0.00	6	−5987.4	32.8	0.00	3	−6556.7	14.8	0.00	3
1948.7	2823.2	0.00	8	−2945.3	199.6	0.00	7	−5541.8	478.4	0.00	8	−6230.9	340.6	0.00	8
−173.3	701.2	0.00	5	−3137.0	7.9	0.01	5	−5918.4	101.8	0.00	5	−6469.7	101.8	0.00	5
104.5	979.0	0.00	7	−2846.2	298.7	0.00	8	−5594.1	426.1	0.00	7	−6242.6	328.9	0.00	7
−331.1	543.4	0.00	4	−3141.6	3.3	0.13	2	−5945.5	74.7	0.00	4	−6497.6	73.9	0.00	4
−74.0	800.5	0.00	6	−3144.9	0.0	0.66	1	−5618.3	401.9	0.00	6	−6254.3	317.2	0.00	6
−872.3	2.2	0.20	3	−3141.5	3.4	0.12	3	−6020.2	0.0	0.92	1	−6571.5	0.0	0.79	1
−872.5	2.0	0.21	2	−3140.6	4.3	0.08	4	−6015.3	4.9	0.08	2	−6568.8	2.7	0.21	2
**Crocodilian nonnative**															
**AIC**	**∆AIC**	***ω***	***r***												
−8476.0	28.8	0.00	4												
−8294.3	210.5	0.00	7												
−8499.3	5.5	0.06	2												
−8345.8	159.0	0.00	6												
−8497.4	7.4	0.02	3												
−8455.1	49.7	0.00	5												
−8504.8	0.0	0.92	1												
–	–	–	–												

The Lomolino regressions displayed sigmoid curves for native amphibians, native frogs, and nonnative turtles, but convex curves for the other taxonomic groups, whereas the Cumulative Weibull regressions displayed sigmoid curves only for native amphibians and native frogs, but convex curves for the rest of the groups (Table 2). Moreover, the Lomolino regressions had a higher probability that an inflexion point occurs within the range of observed island size than the Cumulative Weibull regressions for each taxonomic group (Table 2).

**Table 2:** Shape exploration of the Lomolino and Cumulative Weibull regressions for each taxonomic group. For each model, the fitted parameters (*c*,* z*, and *f*) were obtained using a maximum‐likelihood approach; 95% confidence intervals on the regression parameters as well as the probability of appearance of an inflexion point within the range of observed areas were estimated using a bootstrapping approach

Group	Model^a^	Maximum‐likelihood approach				Bootstrapping approach						
		*c*	*z*	*f*	Shape^b^	*c*		*z*		*f*		Inflexion (%)
						2.5%	97.5%	2.5%	97.5%	2.5%	97.5%	
All species	L	1.4E+6	1.7	3.8E+11	C	3.8E+2	2.6E+7	1.4	5.4E+1	1.2E+4	2.3E+16	19.77
	W	7.5E+7	1.0E−8	5.4E−1	C	3.3E+5	1.2E+10	6.2E−11	4.4E−6	3.5E−1	6.1E−1	0.00
All native	L	2.6E+5	1.8	4.0E+9	C	3.7E+2	2.5E+7	1.4	4.8E+1	1.4E+4	5.2E+17	12.64
	W	1.6E+8	2.2E−9	6.1E−1	C	1.6E+4	7.4E+9	4.1E−11	2.1E−5	3.3E−1	7.1E−1	0.05
All nonnative	L	1.0E+1	2.2	2.7E+2	C	5.5	2.2E+1	1.7	9.3E+3	5.6E+1	4.0E+3	28.20
	W	9.2	1.8E−2	6.8E−1	C	5.4	1.9E+1	1.6E−5	3.5E−2	4.6E−1	1.7	2.15
All amphibian	L	1.8E+2	2.0	1.4E+5	C	1.9E+1	3.1E+5	1.7	2.1E+5	2.2E+3	2.2E+11	24.72
	W	9.6E+1	4.3E−4	7.2E−1	C	7.6E+1	3.1E+8	3.0E−10	1.1E−3	5.2E−1	1.0	2.76
Amphibian native	L	7.7E+1	6.4	1.7E+4	S	2.4E+1	1.3E+5	1.8	7.3E+4	2.8E+3	1.1E+10	54.16
	W	7.5E+1	9.5E−6	1.1	S	7.0E+1	2.0E+8	6.9E−11	3.3E−4	5.8E−1	1.9	33.31
Amphibian nonnative	L	3.6	2.1	4.3E+2	C	1.6	3.6E+1	1.6	6.3E+3	6.1E+1	3.6E+5	22.81
	W	3.6	1.8E−2	5.9E−1	C	1.7	2.8E+2	6.0E−7	3.0E−2	4.6E−1	1.5	2.18
All reptile	L	5.2E+5	1.7	1.1E+11	C	1.6E+2	3.5E+7	1.4	1.3E+2	1.4E+4	1.5E+16	9.82
	W	5.5E+8	1.2E−9	5.4E−1	C	1.0E+2	9.4E+9	6.0E−11	2.5E−2	3.2E−1	6.2E−1	0.00
Reptile native	L	6.8E+5	1.8	3.3E+10	C	3.0E+2	2.6E+7	1.3	6.9E+1	1.6E+4	2.9E+18	7.81
	W	4.6E+8	6.8E−10	6.0E−1	C	5.3E+5	9.2E+9	2.9E−11	1.5E−6	3.0E−1	7.1E−1	0.00
Reptile nonnative	L	7.0	2.2	2.2E+2	C	3.8	1.3E+1	1.7	4.7E+8	5.2E+1	3.1E+3	33.18
	W	6.1	1.8E−2	7.1E−1	C	3.7	1.8E+1	1.3E−8	3.8E−2	4.5E−1	3.6	2.42
Frog	L	1.7E+2	2.0	1.3E+5	C	1.9E+1	3.1E+5	1.7	1.7E+5	2.1E+3	2.4E+11	24.79
	W	9.6E+1	4.2E−4	7.2E−1	C	7.6E+1	3.3E+8	2.5E−10	1.1E−3	5.2E−1	1.0	2.61
Frog native	L	9.2E+1	3.2	2.8E+4	S	2.2E+1	1.5E+5	1.8	8.2E+4	2.7E+3	8.5E+9	53.10
	W	7.5E+1	1.2E−5	1.1	S	7.0E+1	1.8E+8	7.2E−11	3.5E−4	5.7E−1	2.1	33.43
Frog nonnative	L	3.3	2.1	3.8E+2	C	1.6	2.7E+1	1.6	2.1E+4	6.2E+1	1.5E+5	26.37
	W	3.3	1.9E−2	5.9E−1	C	1.6	1.7E+1	3.6E−7	3.0E−2	4.7E−1	2.0	1.58
Turtle	L	2.8	2.4	3.0E+2	C	1.6	4.1	1.7	1.4E+2	1.1E+2	2.6E+3	35.06
	W	2.5	9.1E−3	8.0E−1	C	1.6	3.8	2.2E−4	2.8E−2	4.5E−1	1.4	10.50
Turtle native	L	7.6	1.6	2.5E+5	C	2.3E−3	3.0E+3	9.5E−3	3.0E+110	8.9E+1	6.5E+11	13.57
	W	4.9	5.2E−3	4.5E−1	C	6.6E−1	4.8E+4	1.6E−7	1.2E−2	4.0E−1	1.6	0.05
Turtle nonnative	L	1.4	3.1	1.7E+2	S	4.8E−2	3.0	1.4E−3	8.3E+35	3.4E+1	2.6E+5	60.30
	W	1.4	6.0E−3	9.1E−1	C	6.5E−1	3.1	7.6E−60	5.3E−2	6.0E−1	2.2E+1	7.20
Lizard	L	1.3E+4	1.7	3.2E+8	C	1.8E+2	2.3E+7	1.3	2.8E+2	1.1E+4	3.4E+19	22.36
	W	6.0E+8	9.6E−10	5.1E−1	C	2.1E+2	7.8E+9	4.3E−11	4.8E−4	2.9E−1	1.3	3.82
Lizard native	L	2.1E+4	1.7	4.3E+8	C	1.8E+2	2.1E+7	1.3	3.9E+2	8.7E+3	7.4E+20	27.93
	W	2.4E+4	1.5E−5	5.5E−1	C	2.0E+2	6.6E+9	2.9E−11	1.7E−4	2.7E−1	1.7	10.40
Lizard nonnative	L	5.4	1.7	1.4E+3	C	2.2	2.5E+1	1.4	2.6E+2	6.1E+1	9.9E+5	12.47
	W	5.0	2.9E−2	4.5E−1	C	2.0	1.4E+1	1.9E−5	4.4E−2	3.6E−1	1.6	0.48
Snake	L	4.7E+4	1.5	6.9E+11	C	8.7	1.8E+6	1.5	3.0E+3	2.5E+2	3.5E+15	11.58
	W	1.6E+5	2.0E−6	4.4E−1	C	7.2	9.2E+8	3.2E−10	4.1E−2	4.1E−1	5.7E−1	0.00
Snake native	L	3.4E+5	1.6	1.7E+13	C	7.9	2.3E+6	1.5	5.0E+2	3.4E+2	9.8E+14	12.10
	W	7.7E+8	2.8E−10	4.7E−1	C	7.0	1.3E+9	1.6E−10	4.4E−2	4.3E−1	5.3E−1	0.00
Snake nonnative	L	1.4	2.6	1.7E+2	C	4.7E−1	5.0	1.8	9.9E+130	1.6E+1	6.4E+3	44.72
	W	1.4	1.4E−2	7.4E−1	C	5.4E−1	6.5E+1	3.3E−184	2.6E−2	5.3E−1	8.8E+1	5.05
Crocodilian	L	2.7	1.6	9.7E+3	C	2.8E−2	1.3E+3	3.1E−3	2.7E+15	6.4E+1	4.6E+11	12.23
	W	2.1	1.4E−2	4.6E−1	C	5.7E−1	2.5E+4	5.3E−10	3.0E−2	3.7E−1	3.1	0.63
Crocodilian native	L	2.2	1.6	2.0E+4	C	2.2E−2	6.0E+3	1.0E−3	2.0E+10	2.2E+1	4.0E+14	10.60
	W	1.7	1.6E−2	4.2E−1	C	3.9E−1	3.7E+4	1.6E−7	4.0E−2	3.5E−1	1.4	0.40
Crocodilian nonnative	L	5.9E−1	2.0	2.7E+3	C	9.2E−3	6.3E+2	2.0E−1	2.1E+147	1.9E+1	2.1E+11	17.52
	W	–	–	–	–	1.1E−1	3.8E+3	1.8E−169	1.7E−2	4.0E−1	7.9E+1	3.64

a L refers to Lomolino; W refers to Cumulative Weibull.

b C refers to convex curve; S refers to sigmoid curve.

**Figure 4:**
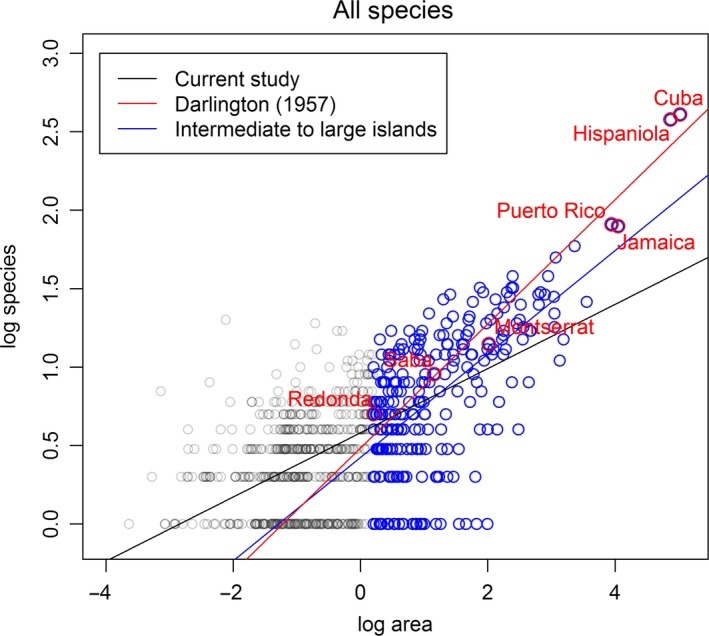
A comparison of the *Z*‐value outcomes among 748 islands conducted in this study, seven islands used by Darlington (1957), and islands of intermediate to large size, showing the inclusion of small islands could lower the *Z*‐value

Darlington (1957) used seven islands to calculate the *Z*‐value of all herpetofauna in this region. However, these seven islands are a subset of intermediate to large islands. In this study, as many small islands were taken into consideration, the *Z*‐value of all herpetofauna was much lower than Darlington's result, suggesting the exclusion of small islands could lead to an overestimation (Figure 4). Here, the *Z*‐values were in the range −0.01 to 0.20 for herpetofauna in the West Indies, and *Z*‐values were higher for increasingly higher taxonomic groups; that is, *Z*‐values for native snake, snake, all reptile, and all species were 0.15, 0.17, 0.19, and 0.2, respectively. We recognized four patterns when comparing *Z*‐values among groups: (1) native > nonnative; (2) reptiles > amphibians; (3) snake > lizard > frog > turtle > crocodilian; and (4) increased from lower‐ to higher‐level taxonomic groups. The *C*‐value of all herpetofauna (0.58) was high as compared with the range 0.524 ± 0.048 for vertebrate studies (Triantis et al., 2012).

**Figure 5:**
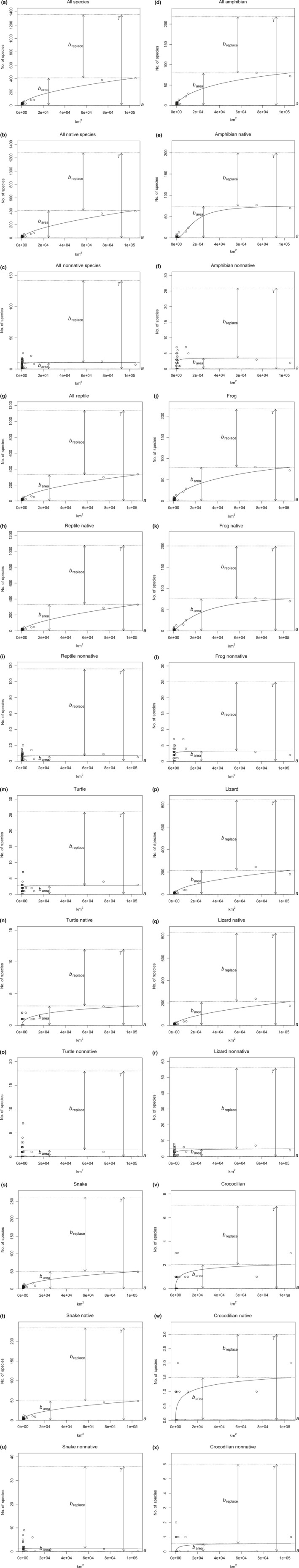
Combination of SAR and additive diversity partitioning, showing species–area curve and *a*,* b*, and γ‐diversity for each taxonomic group. *b* is partitioned into *b*_area_ (contributions made by area) and *b*_replace_ (contributions made by other factors). (a) All species. (b) All native species. (c) All nonnative species. (d) Amphibian. (e) Amphibian native. (f) Amphibian nonnative. (g) Reptile. (h) Reptile native. (i) Reptile nonnative. (j) Frog. (k) Frog native. (l) Frog nonnative. (m) Turtle. (n) Turtle native. (o) Turtle nonnative. (p) Lizard. (q) Lizard native. (r) Lizard nonnative. (s) Snake. (t) Snake native. (u) Snake nonnative. (v) Crocodilian. (w) Crocodilian native. (x) Crocodilian nonnative

According to the additive diversity partitioning for each group (Figure 5), *a* explained only 0.12%–0.67% of the variation in island species richness (mean = 0.25%). On the contrary, *b* explained more than 99% of the variation in island species richness. Thus, in this region and for these taxa, *b *≈ γ‐diversity.

We also compared *b*_area_ and *b*_replace_ for each group (Figure 5). The proportion of *b*_area_ to the total *b* ranged from a low of 3.9% in nonnative snakes to 49.7% in native crocodilians, with an average of 22.8%. The proportion of *b*_replace_ to the total *b* was 77.2% on average, about three times the proportion of *b*_area_.

In the native species groups, the proportion of *b*_area_ to the total *b* ranged from a low of 20.9% in native snakes to 49.7% in native crocodilians, with an average of 32.5%. In the nonnative species groups, the proportion of *b*_area_ to the total *b* ranged from a low of 3.9% in nonnative snakes to 13.7% in nonnative amphibians, with an average of 8.7%. Thus, the area effect played a much less important role in nonnative species than in native taxa.

## 4 Discussion

We found the Cumulative Weibull and Lomolino models were better than the traditional Power and Exponential models to our data. Although Cumulative Weibull and Lomolino are categorized as sigmoid models in the literature (Dengler, 2009; Guilhaumon et al., 2010; Lomolino, 2000; Tjørve, 2009), here we found and proved these two models displayed both convex and sigmoid shapes when applied to different groups (Figure 5; Table 2; Text S1). Our result is consistent with Gentile and Argano (2005), who found the Lomolino model can be convex under some conditions. However, this result is inconsistent with Triantis et al. (2012), who stated the estimated inflexion point of sigmoid regressions often occurs outside the range of observed areas, and thus, the fitted shape is convex in form rather than sigmoid. On the contrary, the fitted convex curves of Cumulative Weibull and Lomolino in our dataset were absolutely convex shape, without inflexion points lying outside the area range. And this conclusion is confirmed through calculations of the first and second derivatives of Lomolino and Cumulative Weibull, through which we further found that assuming all parameters are positive, Lomolino displays sigmoid shape if *z* > *e* and Cumulative Weibull displays sigmoid shape if *f* > 1 (Text S1). So, compared with the traditional Power and Exponential models, the Cumulative Weibull and Lomolino models are superior in that: (1) A J‐shaped portion that may occur at the lower end can explain the minimum area effect that holds that below a certain area threshold, no species would accumulate because there would not be enough area for species to support viable populations (Heatwole & Levins, 1973; Lomolino, 1990; Simaiakis et al., 2012); and (2) their underappreciated flexibility can switch shapes between convex and sigmoid.

Compared with the *C*‐value for the main taxonomic groups summarized by Triantis et al. (2012), the overall *C*‐value in this study was high among vertebrate studies. The high *C*‐value demonstrates that amphibians and reptiles require less space to sustain viable populations than other vertebrates, given the small body size and distinctive physiology of many species. For instance, frogs in the genus *Eleutherodactylus* (Eleutherodactylidae), which comprise the dominant frog fauna of the West Indies, contains terrestrial‐breeding frogs that lay eggs on land or tree leaves, and these eggs later hatch into miniatures of the adults, bypassing the tadpole stage (Hedges, 1993), and that reproductive mode can occur in a cave, on a mountain top, or high in a tree without direct dependency on water; this greatly enhances viability in a water‐deficient and space‐limited environment.

If small islands are excluded from the calculation, *Z*‐value will only represent the SAR from intermediate to large scale. Thereby, the exclusion of small islands could lead to an overestimation of the overall *Z*‐value. Compared with the *Z*‐value ranges pointed by MacArthur and Wilson (1967) and Rosenzweig (1995), the negative value of −0.01 for nonnative crocodilians may due to the small number of islands (*n *=* *9), an inadequate sample size for robust linear modeling (Chase & Bown, 1997). However, the remaining *Z*‐values (0.02–0.20) were also very low.

Over 1,300 species have been recorded in the region, with as many as 407 on the single largest island; however, *a* explained only 0.12%–0.67% of γ‐diversity. The small *a* (0.81) indicates low species evenness, and this is because most of the islands across a large spatial range are small, and over 900 (about half) are not known to support any herpetofaunal species. The low *Z*‐values and small *a* may partly be due to the fact that the “zero” islands (islands with no recorded species) are removed when calculating *Z*‐values but included when calculating *a*. Moreover, the low *Z*‐values and small *a* paradoxically reveal that (1) the reduction in area leads to a significant loss of species especially on small islands, and (2) the low *Z*‐values are an average effect from three different scales, but cannot reflect strong dispersal abilities at all these scales. For instance, native crocodilians have a much lower *Z*‐value than native lizards; however, native crocodilians occupy only 39 islands as compared with 737 for native lizards, indicating *Z*‐value may not be a precise indicator of dispersal ability. Moreover, nonnative species groups have even lower *Z*‐values than native species groups, and this may reveal different species richness scenarios at different scales: First, speciation is impossible for nonnative species on large islands; instead, considerable human activities facilitate the dispersal of nonnative species on intermediate and large islands. The long human presence and high frequency of movements into, out of, and within the region have resulted in many herpetofaunal species functioning as effective dispersers. For instance, the development of the tourist industry often entails the extensive movements of materials (e.g., lumber, decorative plants) among which herpetofaunal species sometimes stowaway (Powell & Henderson, 2012). The trade in live animals (e.g., pets and food) also has played an important role (Perry & Farmer, 2011; Powell et al., 2011). Second, many small islands have no recorded species, reducing the average diversity, whereas other small islands harbor relatively many species, lowering *Z*‐values especially for nonnative species. At least some of these discrepancies likely result from human activities, which continuously supply new colonists to some islands but also transform previous land‐use types into anthropogenic biotopes such as cultivation and settlements (Sfenthourakis, 1996), hosting some herpetofaunal species that coexist with humans (Henderson & Powell, 2001; Raxworthy & Nussbaum, 2000).

The increase of *Z*‐values from lower to higher taxonomic groups is an inevitable result of the positive SARs. Because when a lower taxonomic group is combined with another one, species increments become larger and larger as the increase in island size, leading to a higher resultant *Z*‐value.

Herpetofaunal species may be removed with a reduction in area (Sfenthourakis & Triantis, 2009; Triantis et al., 2006). This situation is particularly detrimental to habitat specialists (Sfenthourakis & Triantis, 2009). Moreover, many very small islands are subject to episodic instability and catastrophic events, such as devastation by storms or inundation by tidal surges, which can lead to total species extinction (MacArthur & Wilson, 1967). Extinction event rather than colonization is thought to be the primary process producing species–area patterns (Losos, 1996; Perry, Rodda, Fritts, & Sharp, 1998; Rand, 1969). However, in this study, we found colonization can still be an important process on small islands, and this conclusion is consistent with that of Russell et al. (2004), who suggested that human activities can be a decisive factor affecting species richness.

Area explained only around 22.8% of the total *b*. The proportion of *b*_replace_ to the total *b* was as high as 77.2%, suggesting rapid within‐island speciation is a main source of new species in this region, for example, the adaptive radiation of anoles (Losos, 2009). Our findings are consistent with those of Losos and Schluter (2000), who indicated that very few species present on small islands cannot also be found on nearby large islands. Apart from within‐island speciation, human introductions, although minor, have become a new mode of entering the region for some species, many of which are not native to the West Indies, enlarging the among‐island heterogeneity. For instance, *Anolis carolinensis* (native to USA) has arrived on Anguilla with the development of tourism (Eaton, Howard, & Powell, 2001).

Area is even weaker at explaining the among‐island heterogeneity for nonnative herpetofaunal species, because nonnative species are introduced via human activities that might be directly caused by the economic development instead of island area (Powell et al., 2011). Normally, the larger the island, the larger human population and richer natural resources there would be, so economic development may have a positive correlation with island size. However, this is not always the case. For example, the US–Cuban trade embargo strongly increases Cuban economic isolation (Helmus et al., 2014), lowering the estimated *S*_max_ and therefore decreasing area effect toward explaining the among‐island heterogeneity for nonnative species. On the other hand, larger islands have lower extinction rate, higher colonization, and more in situ speciation than smaller islands, maximizing the estimated *S*_max_ for native species.

Suitable herpetofaunal habitats are deteriorating on many (maybe most) islands as a consequence of human activities (Perry & Farmer, 2011; Powell & Henderson, 2012). Consequently, the probability of alien species becoming established is increased, and Powell et al. (2011) strongly recommended that an early detection and monitoring system for alien species be established in this region. If current trends continue, as in other regions of the world (e.g., Perry et al., 1998; Whitaker, 1973), we suggest that a number of endemic West Indian herpetofaunal species will be extirpated from large islands and might survive solely (if at all) on tiny offshore islets. A *Z*‐value of around 0.3 obtained by Darlington (1957) suggests if the area is reduced by 90%, then the number of species it supports will be halved. However, our study demonstrated that *Z*‐value is scale dependent, so the loss rate of species against area reduction is also scale dependent rather than constant across scales, and native species on large islands are most sensitive to area reduction. Thereby, we also recommend that conservation priority should be given to large and habitat‐rich islands with the most species‐rich community to maximize the number of endemic species preserved.

## 5 Conclusions

The Cumulative Weibull and Lomolino models are reported to be sigmoid (Dengler, 2009; Guilhaumon et al., 2010; Lomolino, 2000; Simaiakis et al., 2012; Tjørve, 2009). However, in this study, we found that they can display both convex and sigmoid curves, suggesting their flexibility has been underappreciated. *Z*‐values may be overestimated if small islands are excluded from calculation. Species diversity is generated among islands, and the high proportion of *b*_replace_ to total *b* suggests that within‐island speciation rather than island area is the main source of new native species in this region.

The complex geological history of the West Indies offers many opportunities for dispersal and vicariance to affect biotas (e.g., Donovan & Jackson, 1994; Hedges, 2001). Also, human activities are having a profound impact on local biotas, including the herpetofaunas. The comparisons of *Z*‐values and the proportion of *b*_area_ to total *b* between native and nonnative species reflect human activities accelerating the rate of over‐water dispersal and weakening the area effect within the region. The contrast between small *a* and low *Z*‐values paradoxically reveals the following: (1) The reduction in area leads to a significant loss of species especially on small islands; (2) there are both extinctions and increases in *b* on small islands under natural and human‐mediated conditions, respectively; and (3) many herpetofaunal species have strong over‐water dispersal ability especially on islands of intermediate size. Human activities can temporarily enhance species richness on small islands, but also can disturb habitats and introduce predators and competitors, consequently increasing the extirpations of populations and disrupting the complex but often fragile communities on large islands.

## Conflict of Interest

None declared.
