# Supplementary material for: Species–area relationships and additive partitioning of diversity of native and nonnative herpetofauna of the West Indies
Source: Ecol Evol. 2016 Oct 5;6(21):7742–62. doi: 10.1002/ece3.2511 (PMC6093157; doi:10.1002/ece3.2511)
Supplement: Supplementary file 1 [file ECE3-6-7742-s001.docx]

**Text S1.** Calculations of the first and second derivatives of Lomolino and Cumulative Weibull to test the shapes of all native species, native reptiles, and native amphibians.

***First derivative of Lomolino*:**

$$S^{'}=\frac{-cz^{\log\left(f/A\right)}\left(-1/A\right)\log z}{\left(1+z^{\log\left(f/A\right)}\right)^{2}}$$

$$=\frac{cz^{\log\left(f/A\right)}\log z}{A\left(1+z^{\log\left(f/A\right)}\right)^{2}}$$

***Second derivative of Lomolino:***

$$S^{''}=c\log z\left[\frac{z^{\log\left(f/A\right)}}{A\left(1+z^{\log\left(f/A\right)}\right)^{2}}\right]^{'}$$

$$=c\log z\left[\frac{\left(z^{\log\left(f/A\right)}\right)^{'}A\left(1+z^{\log\left(f/A\right)}\right)^{2}-z^{\log\left(f/A\right)}\left[A\left(1+z^{\log\left(f/A\right)}\right)^{2}\right]^{'}}{A^{2}\left(1+z^{\log\left(f/A\right)}\right)^{4}}\right]$$

$$=c\log z\left[\frac{z^{\log\left(f/A\right)}\left(-1/A\right)A\left(1+z^{\log\left(f/A\right)}\right)^{2}\log z-z^{\log\left(f/A\right)}\left[\left(1+z^{\log\left(f/A\right)}\right)^{2}+2A\left(1+z^{\log\left(f/A\right)}\right)z^{\log\left(f/A\right)}\left(-1/A\right)\log z\right]}{A^{2}\left(1+z^{\log\left(f/A\right)}\right)^{4}}\right]$$

$$=c\log z\left[\frac{-z^{\log\left(f/A\right)}\left(1+z^{\log\left(f/A\right)}\right)^{2}\log z-z^{\log\left(f/A\right)}\left[\left(1+z^{\log\left(f/A\right)}\right)^{2}-2\left(1+z^{\log\left(f/A\right)}\right)z^{\log\left(f/A\right)}\log z\right]}{A^{2}\left(1+z^{\log\left(f/A\right)}\right)^{4}}\right]$$

$$=-\frac{cz^{\log\left(f/A\right)}\left(1+z^{\log\left(f/A\right)}\right)\log z}{A^{2}\left(1+z^{\log\left(f/A\right)}\right)^{4}}\left[\left(1+z^{\log\left(f/A\right)}\right)\log z+\left(1+z^{\log\left(f/A\right)}\right)-2z^{\log\left(f/A\right)}\log z\right]$$

$$=-\frac{cz^{\log\left(f/A\right)}\log z}{A^{2}\left(1+z^{\log\left(f/A\right)}\right)^{3}}\left[\log z+1+z^{\log\left(f/A\right)}-z^{\log\left(f/A\right)}\log z\right]$$

$$=-\frac{cz^{\log\left(f/A\right)}\log z}{A^{2}\left(1+z^{\log\left(f/A\right)}\right)^{3}}\left[\log z+1+z^{\log\left(f/A\right)}\left(1-\log z\right)\right]$$

The positive or negative nature of $S^{''}$is determined by $\log z+1+z^{\log\left(f/A\right)}\left(1-\log z\right)$, because $\frac{cz^{\log\left(f/A\right)}\log z}{A^{2}\left(1+z^{\log\left(f/A\right)}\right)^{3}}$is always positive in our case. For all native species and native reptile species groups of experiment results of *c*, *z*, and *f* (Table 2), we have $\log z=0.59$(base = *e*),$1-\log z>0$, and $\log z+1>0$ which means $\log z+1+z^{\log\left(f/A\right)}\left(1-\log z\right)>0$. So$S^{''}<0$, which means the graph of $S$ is concave down.

But for native amphibian species group, $\log z=1.85$(base = *e*), so$1-\log z<0$. Then we substitute the parameters value into $\log z+1+z^{\log\left(f/A\right)}\left(1-\log z\right)$and get: if$A=8886.65$(km^2^), $S^{''}=0$; if$A>8886.65$(km^2^),$S^{''}<0$; and if $A<8886.65$(km^2^), $S^{''}>0$. That means the graph of $S$ is sigmoid shape.

***First derivative of Cumulative Weibull:***

$$S^{'}=-c\exp(-\mathrm{zA}^{f})(-z)\mathrm{fA}^{f-1}$$

$$\begin{matrix} =czf\exp(-\mathrm{zA}^{f})\mathrm{A}^{f-1} \\ \end{matrix}$$

***Second derivative of Cumulative Weibull:***

$$S^{''}=czf\left[\left(\exp(-\mathrm{zA}^{f})\right)^{'}\mathrm{A}^{f-1}+\exp(-\mathrm{zA}^{f})(\mathrm{A}^{f-1})^{'}\right]$$

$$=czf\left[\exp(-\mathrm{zA}^{f})(-\mathrm{z})\mathrm{fA}^{f-1}\mathrm{A}^{f-1}+\exp(-\mathrm{zA}^{f})(\mathrm{f}-1)\mathrm{A}^{f-2}\right]$$

$$=czf\left[-\mathrm{zfexp}(-\mathrm{zA}^{f})\mathrm{A}^{2f-2}+\exp(-\mathrm{zA}^{f})(\mathrm{f}-1)\mathrm{A}^{f-2}\right]$$

$$=czf\mathrm{A}^{f-2}\exp(-\mathrm{zA}^{f})\left(-\mathrm{zfA}^{f}+\mathrm{f}-1\right)$$

The positive or negative nature of $S^{''}$is determined by $-\mathrm{zfA}^{f}+\mathrm{f}-1$, because $czf\mathrm{A}^{f-2}\exp(-\mathrm{zA}^{f})$is always positive in our case. For all native species and native reptile species groups of experiment results of $c$, $z$, and $f$, we have $\mathrm{f}-1<0$and $-\mathrm{zfA}^{f}<0$ which means $-\mathrm{zfA}^{f}+\mathrm{f}-1<0$. So$S^{''}<0$, which means the graph of $S$ is concave down.

But for native amphibian species group, $\mathrm{f}-1>0$. Then we substitute the parameters value into $-\mathrm{zfA}^{f}+\mathrm{f}-1$and get: if$A=4158.96$(km^2^), $S^{''}=0$; if$A>4158.96$(km^2^),$S^{''}<0$; and if $A<4158.96$(km^2^), $S^{''}>0$. That means the graph of $S$ is sigmoid shape.

In summary, Lomolino and Cumulative Weibull displayed convex shape in all native species and native reptile species groups, but displayed sigmoid shape in native amphibian species group. Assuming all parameters are positive, Lomolino can display sigmoid shape if$z>e$ and Cumulative Weibull can display sigmoid shape if$f>1$.
